# Early-Life Family Structure and Microbially Induced Cancer Risk

**DOI:** 10.1371/journal.pmed.0040007

**Published:** 2007-01-16

**Authors:** Martin J Blaser, Abraham Nomura, James Lee, Grant N Stemmerman, Guillermo I Perez-Perez

**Affiliations:** 1 Department of Medicine, New York University School of Medicine, New York, New York, United States of America; 2 Department of Microbiology, New York University School of Medicine, New York, New York, United States of America; 3 Veterans Administration Medical Center, New York, New York, United States of America; 4 Kuakini Medical Center and University of Hawaii, Honolulu, Hawaii, United States of America; 5 University of Cincinnati, Cincinnati, Ohio, United States of America; Institutionen for Medicinsk Epidemiologi och Biostatistik, Sweden

## Abstract

**Background:**

Cancer may follow exposure to an environmental agent after many decades. The bacterium *Helicobacter pylori,* known to be acquired early in life, increases risk for gastric adenocarcinoma, but other factors are also important. In this study, we considered whether early-life family structure affects the risk of later developing gastric cancer among H. pylori ^+^ men.

**Methods and Findings:**

We examined a long-term cohort of Japanese-American men followed for 28 y, and performed a nested case-control study among those carrying H. pylori or the subset carrying the most virulent *cagA^+^ H. pylori* strains to address whether family structure predicted cancer development. We found that among the men who were *H. pylori^+^* and/or *cagA* ^+^ (it is possible to be *cagA^+^* and H. pylori ^−^ if the H. pylori test is falsely negative), belonging to a large sibship or higher birth order was associated with a significantly increased risk of developing gastric adenocarcinoma late in life. For those with *cagA^+^* strains, the risk of developing gastric cancer was more than twice as high (odds ratio 2.2; 95% confidence interval 1.2–4.0) among those in a sibship of seven or more individuals than in a sibship of between one and three persons.

**Conclusions:**

These results provide evidence that early-life social environment plays a significant role in risk of microbially induced malignancies expressing five to eight decades later, and these findings lead to new models to explain these interactions.

## Abstract 2

This study suggests that early-life social environment has a significant role in risk of microbially induced malignancies such as gastric adenocarcinoma occuring five to eight decades later.

## Editors' Summary

**Background.:**

Although the theory that certain cancers might be caused by infectious agents (such as bacteria and viruses) has been around for some time, concrete evidence linking specific cancers and infections is only recently beginning to emerge. There is now very good evidence that stomach cancer, once one of the frequent types worldwide but now less common, is strongly associated with a particular infection of the stomach lining. This specific bacterium colonizing the stomach, Helicobacter pylori (or H. pylori), often infects people early in childhood through close contact with other people, and tends to stay in the body throughout life. However, most people do not suffer any symptoms as a result of being colonized with *H. pylori.* Researchers are interested in the relationship between stomach cancer and aspects of someone's upbringing, for example whether an individual has a large number of sisters and brothers and whether they are the youngest or oldest in a large group of siblings. One reason for being interested in this topic is that if H. pylori is mainly spread from one child to another in the home, we might expect children from large sibling groups, and the youngest children in a group, to be at greater risk of being infected, and then more likely to get stomach cancer later in life. Furthermore—and this was the primary reason for the study—the researchers wished to determine whether, among *H. pylori^+^* people, the structure of the family affects the risk of developing stomach cancer much later in life. With all study participants being *H. pylori^+^*, the essential comparison was between people of high and low birth order.

**Why Was This Study Done?:**

This group of researchers had already done a previous study that had shown that people who carry *H. pylori* *H. pylori* in their stomachs are more likely to get stomach cancer, and also that younger children in a sibling group are more likely to get stomach cancer. In the period following that study, the examined population has become older and more of the people concerned have developed stomach cancer. This meant that the researchers could go back and extend their previous work to see, more reliably, whether stomach cancer was linked to family structure. It also meant that the researchers could look at the effects of each factor not only in isolation, but also the combined effect of all the different factors. The researchers also stratified for the most virulent strains (those that were *cagA^+^*).

**What Did the Researchers Do and Find?:**

In this study, the researchers started out with a pool of 7,429 Japanese-American men living in Hawaii, USA, who had donated blood samples between 1967 and 1975. Of these men, 261 eventually developed stomach cancer. Each of the 261 men was then matched with a similarly aged man from the original pool of 7,429 men who did not have stomach cancer. The researchers then went back to the original blood samples taken many years before and tested the samples to see if the men were infected with H. pylori at the time the sample was taken and, if so, whether a particular strain of the bacterium, *cagA,* was present. The researchers then looked at whether the risk of getting stomach cancer was associated with the number of siblings a man had and whether he was older or younger than the other siblings.

Similar to the prior study, they found that men who had stomach cancer were three times more likely to carry H. pylori compared to men who did not develop stomach cancer. In men who had *H. pylori,* those with large numbers of siblings were more likely to get stomach cancer, and this was especially true for men who had the *cagA* strain of *H. pylori.* In the whole group of men with cancer, the order of birth (whether a man was older or younger in his sibling group) did not seem to be particularly linked to development of stomach cancer. However, in men who had the *cagA* strain of *H. pylori,* those from the largest sibships were at highest risk of developing gastric cancer; in this group, one particular type of cancer (the most common type—intestinal-type gastric cancer) was also associated with later birth order.

**What Do These Findings Mean?:**

The researchers initially thought that men with H. pylori would be at a higher risk of getting stomach cancer if they had a large number of sisters and brothers, and especially if they were a younger sibling in a large group. This idea was supported by their data. These findings support the idea that people often get H. pylori from their older sisters and brothers, but there is not conclusive proof of this. There might be some other factor that explains the association between large family size and stomach cancer, for example that people from large families might be poorer and more at risk from stomach cancer for some other reason. Currently, most doctors do not recommend routinely testing people without any symptoms to see if they have *H. pylori,* but people with pain or discomfort in the upper abdomen would generally be screened for H. pylori and then treated to eliminate the infection if it is found. The main novel idea is that those people who are born in a large sibship, and/or are of higher birth order, are more likely to acquire their H. pylori from a genetically related person (a sibling) than from an unrelated person (friend/classmate). This “family-structure effect” could be the explanation as to why there is a higher risk of stomach cancer developing later—the strain from a genetically related person already is “preadapted” to the new host, and has a “head-start” on immunity, compared to a strain from an unrelated person. The researchers hypothesize that it is the nature of that initial interaction with the host that sets the stage for the kind of events that lead to cancers decades later.

**Additional Information.:**

Please access these Web sites via the online version of this summary at http://dx.doi.org/10.1371/journal.pmed.0040007.

- A Perspective article by Dimitrios Trichopoulos and Pagona Lagiou discusses these findings further
- MedLine Plus encyclopedia entry on stomach cancer
- Wikipedia entry on Helicobacter pylori (Wikipedia is an internet encyclopedia that anyone can edit)
- The US National Cancer Institute publishes information about stomach cancer

## Introduction

Cancer is generally a disease of old age with mortality rates increasing with each succeeding decade of life [1]. However, for many forms of cancer, the relevant risks are conferred by exposure to environmental agents decades earlier [2,3]. Long latencies between exposure to a carcinogen and clinical expression of malignancy occur with asbestos and mesothelioma, cigarette smoking and lung cancer, and hepatitis B virus and hepatoma [4]. Adenocarcinoma of the stomach, one of the most common causes of cancer death in the world, clearly follows this pattern [5].

Carriage of the gram-negative gastric bacterium, *Helicobacter pylori,* increases the risk for development of gastric adenocarcinoma [6–8]. H. pylori is predominantly acquired in childhood, most commonly after the first year of life [9]; perinatal transmission is uncommon. Family size and birth order affect transmission; later-born children from large sibships are at greatest risk for acquiring the organism [10,11]. Such observations suggest that sibling-to-sibling transmission is critical, and the progressive disappearance of H. pylori seen with socioeconomic development [12,13] is consistent with falling family sizes. For hepatitis B virus, birth order influences risk of disease [14,15], and for measles, viral acquisition from a family member increases disease severity [16]. Studies of migrants have shown that early life is the window for the critical environmental exposure that heightens gastric cancer risk [17]; the most important environmental risk factor for gastric cancer is acquisition of H. pylori [6–8]. Since early-life family structure affects microbial transmission, we hypothesized that it would influence risk of H. pylori-associated gastric cancer. Consistent with this notion has been the repeated observation that gastric cancer risk is related to the number of siblings during childhood [18–20].

H. pylori strains can be characterized by the presence or absence of the *cag* island, a 35–40-kb chromosomal region containing type IV secretion system genes that encode proteins that inject the CagA protein into the host epithelium [8]. The *cagA* status of the H. pylori cells colonizing a host can be determined by the presence of serum IgG antibodies to the CagA protein [21,22]. Compared with *cagA* ^−^ strains, carriage of *cagA^+^ H. pylori* strains is associated with increased risk of both premalignant lesions and gastric cancer [21,23–27].

Our hypothesis was that among *H. pylori^+^* (and *cagA* ^+^) men, early-life family structure (belonging to a larger sibship or being of higher birth order) enhanced the risk for gastric cancer development decades later. We tested the hypothesis as part of nested case-control studies of Japanese-American men in Hawaii that have been conducted to define risk factors for developing gastric cancer in this well-defined high-risk population group [20,21,26,28]. In analyses of 109 men who developed gastric cancer over a 21-y observation period and their matched controls from this cohort, we have previously shown that H. pylori positivity [28], CagA seropositivity [21], and higher birth order [20] each are associated with gastric cancer risk.

In the current investigation, we studied 261 men (including the earlier 109) who developed incident gastric cancer over a 28-y observation period [26], to more precisely define the association of early-life family structure, and *H. pylori cagA* status, with risk of developing gastric cancer decades later.

## Methods

### Study Population

Serum samples were available on 7,429 Japanese-American men who were examined between 1967 and 1975, as described [26,28]; the 6,860 men who were reexamined from 1971 to 1975 also were asked to name their brothers, if they had a brother. Since between 1975 and 1977, 2,534 (66%) of the 3,843 identified brothers provided a blood sample, a total of 9,963 men (7,429 + 2,534) were included in the study. The discharge records of all general hospitals on Oahu were monitored to identify cases of stomach cancer occurring among the examined men during the study. Twenty-eight patients who were diagnosed with gastric cancer before their phlebotomy were excluded from the study. Among the remaining cohort of 9,935 men, 279 incident cases of gastric carcinoma were diagnosed from 1968 to 1996; each case was confirmed by examination of tissue obtained at surgery or by biopsy [26,28]. Among these 279 incident cases, there were 18 patients with cancer of the cardia, defined as such if the cardioesophageal junction was involved, who also were excluded from the study. In all, 217 non-cardia gastric cancer cases were diagnosed among the 7,429 original cohort men and 44 cases were diagnosed among the brothers, for a total of 261 cases [25]. The mean (± standard deviation) age at cancer diagnosis was 72.7 ± 8.0 y [26]. On entry into the cohort, each man was asked to enumerate their older and younger brothers and sisters, permitting sibship size and birth order to be determined. For their brothers, only sibship size was available; thus, for the birth-order part of the study, the number of cases and matched controls is 217. The histologic type of carcinoma was determined according to the classification of Lauren [29]. For the total group, there were 184 cases of intestinal cancer, 49 cases of diffuse cancer, 21 cases of mixed-type cancer, and seven cases whose type was unknown.

### Selection of Control Participants

Each case patient was matched with one control participant from the study cohort according to age at examination and date of serum collection. If a potential control participant had a gastrectomy before the serum was obtained or had been given a diagnosis of peptic ulcer disease, according to hospital records, before or after the serum was obtained, he was excluded from the study, because of the reported association between H. pylori and peptic ulcer disease [28]. As a result, 774 potential control participants (8%) were removed from the pool of 9,656 men without gastric cancer. Although excluding peptic ulcer disease cases from the potential control group may possibly increase the magnitude of the association of H. pylori and gastric cancer, this association is now well recognized [6–8], and it should have no effect on the hypotheses tested in this study unless there were substantial differences in family structure. Of the 261 case-control pairs identified, each pair was born within 1 y of each other, except for three pairs (median difference of 2.0 y), and each pair was examined within 1 mo of each other, except for 35 pairs (median difference of 3 mo). Each control participant was alive and did not have any cancer diagnosis at the time of diagnosis of the matched case; therefore, death was not a competing risk in this study. For the 44 brothers who were case patients, their controls came from the pool of brothers and were matched according to the same criteria. Since both the cases involving brothers and the control participants were brothers of the original cohort members, there is no a priori bias for family size.

### Serologic Methods

The frozen sera were shipped in dry ice for analysis; the testing laboratory was blinded as to the case or control status associated with the specimens and treated them identically in the analysis. H. pylori antibody status was determined by IgG ELISA using whole-cell sonicates from a pool of H. pylori strains, as described [26,28]. The sensitivity and specificity of the methodology used have been determined to be 98% and 91% [30], respectively. IgG antibodies to a recombinant 66-kDa fragment of CagA cloned in E. coli cells were detected by ELISA, as described [21,26]. The sensitivity and specificity of this assay were determined to be 94% and 93%, respectively [21]. In a previous evaluation of these assays, using blinded quality-control samples, the mean, standard deviation, and coefficient of variation were 1.83%, 0.26%, and 14.2%, respectively, for the whole-cell assay and 0.15%, 0.04%, and 26.7%, respectively, for the CagA assay [31]. H. pylori positivity was defined as positivity in either the H. pylori or CagA assay, as described [22].

### Statistical Analysis

The risk of gastric cancer and its two histologic types (intestinal and diffuse) associated with sibship size and birth order was assessed by the odds ratios and confidence intervals (CIs) estimated by using logistic regression modeling [32]. Conditional logistic regression was used when the age-matched case-control pairs were maintained, and unconditional logistic regression was used when the age-matched case-control pairs were not maintained, as described [20]. Each exposure variable (sibship size or birth order) was categorized into three groups to create a set of binary indicator variables with the lowest category as the reference group. The groupings for birth order (1, 2–3, ≥4) were exactly as in our prior study [20]. For analysis of sibship-size, groupings (1–3, 4–6, ≥7) were selected to have roughly equivalent numbers in each group. These indicator (exposure) variables and other confounding covariates (smoking history and age, in unconditional logistic regression) were used as explanatory variables in the model for the estimation of odds ratios, because they had been associated with gastric cancer in this cohort [20]. The test for trend was performed using the three class mid-points of sibship size or birth order as explanatory variables, and the score statistic [33] was used to determine statistical significance. All *p*-values and CIs are based on two-tailed tests. Statistical analyses were performed with SAS software (SAS Institute, http://www.sas.com) [34].

## Results

On entry into the study cohort, 239 (92%) of the 261 men who later developed gastric cancer carried *H. pylori,* and 189 (72%) carried *cagA* ^+^ strains (Table 1). In comparison, 205 (79%) of the 261 matched controls carried H. pylori and 155 (59%) carried *cagA* ^+^ strains. These differences pointed toward associations between gastric cancer and H. pylori (odds ratio = 2.97 [95% CI = 1.70–5.21]) and *cagA* ^+^ H. pylori strains (odds ratio = 1.80 [95% CI = 1.22–2.64]). In a prior analysis, the presence of H. pylori (odds ratio = 2.7 [95% CI = 1.3–5.6]), and specifically *cagA* ^+^ strains (odds ratio = 4.1 [95% CI = 2.2–7.7]), was associated with increased risk of developing intestinal-type gastric cancer during the 28-y observation period [26].

**Table 1:**
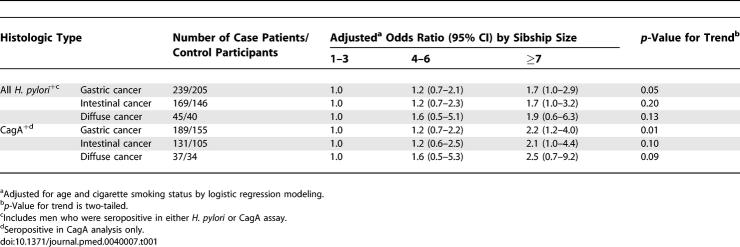
Adjusted Odds Ratios of Sibship Size on Gastric Cancer Risk, Stratified by Histologic Type and by H. pylori and CagA Seropositivity

Examination of the entire group showed no statistically significant association of sibship size or birth order with gastric cancer risk (Table S1). Adenocarcinomas of intestinal or diffuse type were present in 184 (70%) and 49 (19%) of patients, respectively; in the other patients, the tumors were of mixed type (*n* = 21) or were not classified (*n* = 7). The primary analysis of this study examined only those patients and control participants carrying *H. pylori,* or more specifically *cagA^+^ H. pylori* strains, so as to control for these important and previously known [18,21–25] risk factors. We focused on the intestinal and diffuse types, which are the best defined and most common histological forms of gastric cancer worldwide [29], as well as in this population [26,28].

Among the H. pylori ^+^ men, those born in the largest sibships were at statistically significantly increased risk for developing gastric cancer (Table 1). Among men carrying *cagA^+^ H. pylori* strains, those from the largest sibships were at greatest risk of developing gastric cancer. In both the H. pylori and *cagA* analyses, there were statistically significant trends associating gastric cancer risk with sibship size. Twenty-five men who had gastric cancer that was not intestinal or diffuse were excluded from the subtype analyses. Men from larger sibships had increased risk of developing either intestinal or diffuse-type cancer, but most differences were not statistically significant. Among the men carrying any H. pylori strains (Table 2), development of intestinal but not diffuse-type gastric cancer was associated with higher birth order. For men with *cagA* ^+^ strains, the trend associating increasing risk of developing intestinal-type gastric cancer with later birth order was progressive and statistically significant.

**Table 2:**
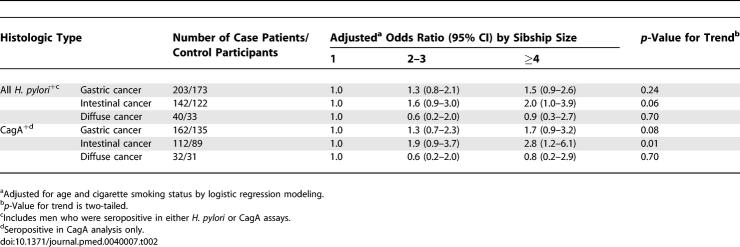
Adjusted Odds Ratios of Birth Order on Gastric Cancer Risk, Stratified by Histologic Type and by H. pylori and CagA Seropositivity

## Discussion

In this study, we found that among men carrying *H. pylori,* especially *cagA^+^* strains, the risk of developing gastric cancer (particularly of the intestinal type) was greatest among those from large sibships or with higher birth order (which covary). Men carrying *H. pylori,* especially *cagA^+^* strains, have the highest risk of developing gastric cancer later in life compared with men without H. pylori [21,24–26]. We previously addressed the question of early-life family structure and gastric cancer risk [20], but the present study extends that analysis by including more than double the number of participants, longer follow-up (28 y), and stratification for *cagA* status, which now is recognized as a principal H. pylori virulence factor [35] that increases gastric cancer risk [20–24]. Our study included analyses of cohort members and their brothers. Could this have introduced a bias? However, there were only 11 pairs of brothers in which both were case patients, no pairs of brothers in which both were control participants, and one pair of brothers in which one was a case patient and the other was a control participant. There were no instances in which three or more brothers were included in the study. When the generalized estimating equations modeling approach was used to correct for intracluster correlation, the analysis, as expected, produced virtually identical results (unpublished data) as compared to the results by logistic regression modeling, which does not correct for intra-cluster correlation.

Another limitation of the study is exclusion of patients with peptic ulcer disease from the control group but not from the case group, and the possibility that this might introduce a bias. In our earlier study [20], there was no association between duodenal ulcer and birth order or sibship size, or between gastric ulcer and sibship size. However, gastric ulcer was associated with birth order. Since about 8% of the control participants had been removed from the case group, and gastric ulcer represents about 70% of all peptic ulcers in that population [36], we estimate that <6% had gastric ulcers, so their exclusion from the pool of control participants most likely had a minimal effect on the results of the analysis.

For this high-risk group (*H. pylori^+^ cagA* ^+^), our observations show that early-life family structure is associated with a differential risk of developing gastric cancer, which chiefly presents seven or eight decades later. Although our a priori hypothesis predicted this result, we used the conservative two-tailed analysis to seek the most significant associations; use of a one-tailed analysis would not change the overall trends, but would increase the statistical significance of the findings. Our findings are most applicable to the intestinal type of gastric cancer [7,26,28,29], which is the most common, since our study population permits a more robust analysis than for the diffuse or indeterminate types of cancer. Since our study examined men only, and of a particular ethnic and socioeconomic group, we are limited in generalizing to the population at large. However, the relative homogeneity of the group reduces unidentified confounding that might interfere with detecting real findings.

The results are internally consistent, and support the hypothesis that among H. pylori (especially *cagA*)–positive persons, variation in early-life family structure affects risk of gastric cancer. The associations with large sibships and with later birth order could imply that the affected individuals acquired the relevant H. pylori strain as a child from an older sibling. Alternatively, large sibships may be a marker for low socioeconomic status, and transmission still could be primarily mother to child. However, the range of socioeconomic status in this population was narrow [26], and infected older siblings are known to be important vectors for H. pylori transmission [10,11]. Nevertheless, both of these possibilities further imply either early-life acquisition of one or multiple H. pylori strains, and/or acquisition of an H. pylori strain that had been preadapted to a family member. That such a difference could affect risk of a malignancy that first manifests 50–80 y later is a striking finding, and is a paradigm for other late-in-life malignancies and degenerative diseases [2,3]. Conversely, high birth order and large sibship have an opposite association (decreased risk) with Hodgkin's disease [37], and potentially with childhood leukemia [38,39]. Hodgkin's disease has a peak incidence between 20 and 29 y of age, and the family structure data have been interpreted as indicating that early-born children have delayed acquisition of a commonly circulating pathogen (possibly Epstein-Barr virus) compared to late-born children [38].

One mechanism for the age-specific differences is that the immune system continues to mature during childhood [40]. Thus, immune responses of hosts of different ages may qualitatively (and quantitatively) differ after exposure to a particular microbe, as is well established for *Varicella zoster,* hepatitis A and B, and Epstein-Barr virus infections [41]. Alternatively, an organism that is “preadapted” to a new host may have a substantial advantage vis-à-vis the host immune response. Microbial genomes are plastic, and H. pylori is especially so [42–44]. Passage through a host selects for particular genotypes [45], and the spreading of these genotypes to a genetically related host may lead to a better adapted [46], and thus more virulent, bacterial population than occurs when transmitted from an unrelated individual. An alternative hypothesis is that the observed phenomena reflect as-yet-unidentified cofactors associated with high birth order and large sibships; candidate factors include multiple H. pylori strains [47], other childhood infections, and stresses associated with large sibships [2]. Although early acquisition increases the total length of colonization of the host by *H. pylori,* the effect of a difference of only a few years may be of much smaller magnitude than the effects from the above phenomena, occurring at possibly critical developmental steps.

In summary, the decline in gastric cancer observed during modernization across developed nations [1,5] may reflect not only the progressive disappearance of H. pylori [12], especially *cagA^+^* strains [13], but, as transmission becomes less intense, this may reflect a change in the average age of acquisition [11], in the number of different strains acquired [47], or in their preadaptation to particular hosts [42,43]. As such, the interaction of H. pylori with gastric cancer development is important per se, and is also important as a model system for other malignancies or chronic diseases in which microbial persistence plays a pathogenic role.

## Supporting Information

Table S1 Adjusted Odds Ratios of Sibship Size and Birth Order on Gastric Cancer Risk, Stratified by Histologic Type (23 KB DOC)

## Abbreviation

CI: confidence interval
