# Correction: Early-Life Family Structure and Microbially Induced Cancer Risk

**DOI:** 10.1371/journal.pmed.0040100

**Published:** 2007-02-27

**Authors:** Martin J Blaser, Abraham Nomura, James Lee, Grant N Stemmerman, Guillermo I Perez-Perez

In *PLoS Medicine*, volume 4, issue 1: doi:10.1371/journal.pmed.0040007

Table 2 included a column entitled “Adjusted Odds Ratio (95% CI) by Sibship Size.” This should have read “Adjusted Odds Ratio (95% CI) by Birth Order.”

