# Supplementary material for: Early-Life Family Structure and Microbially Induced Cancer Risk
Source: PLoS Med. 2007 Jan 16;4(1):e7. doi: 10.1371/journal.pmed.0040007 (PMC1769414; doi:10.1371/journal.pmed.0040007)
Supplement: Supplementary file 1 [file pmed.0040007.st001.doc]

**Online Supplementary Table S1**

**Adjusted odds ratios of sibship size and birth order on**

**gastric cancer risk, stratified by histologic type**

A. Sibship size

| Histologic  type | Number of case/controls | Adjusted* Odds ratio (95%  confidence intervals) by sibship size | | | p-value  for  trend** |
| --- | --- | --- | --- | --- | --- |
|  |  | 1-3 | 4-6 | 7+ |  |
| Gastric cancer | 261/257 | 1.0 | 1.3 (0.8-2.2) | 1.4 (0.8-2.4) | 0.54 |
| Intestinal cancer | 184/180 | 1.0 | 1.4 (0.7-2.7) | 1.5 (0.8-2.8) | 0.92 |
| Diffuse cancer | 49/49 | 1.0 | 1.6 (0.5-4.9) | 1.5 (0.4-6.2) | 0.24 |

B. Birth order

| Histologic  type | Number of case/controls | Adjusted* Odds ratio (95%  confidence intervals) by birth order | | | p-value  for  trend*** |
| --- | --- | --- | --- | --- | --- |
|  |  | 1 | 2-3 | 4+ |  |
| Gastric cancer | 217/213 | 1.0 | 1.1 (0.7-1.8) | 1.3 (0.7-2.2) | 0.91 |
| Intestinal cancer | 152/148 | 1.0 | 1.4 (0.8-2.5) | 1.6 (0.8-3.1) | 0.45 |
| Diffuse cancer | 41/41 | 1.0 | 0.5 (0.2-1.7) | 0.7 (0.2-2.9) | 0.57 |
| *Adjusted for age and cigarette smoking status by logistic regression modeling  **p for trend is 2-tailed | | | | | |
